# *Fusarium awaxy* Associated with Maize from Paraguay: A First Report

**DOI:** 10.3390/jof11110794

**Published:** 2025-11-07

**Authors:** Guillermo Andrés Enciso-Maldonado, Fernando Jesús Lugo-Pedrozo, Gabriela Micaela Romero, Rosana Vázquez, Lorena Liebl-Meza, Silverio Andrés Quintana-Arrúa, María Laura Ramírez, Eugenia Cendoya, Danilo Fernández Ríos, Marco Maidana-Ojeda, Andrea Alejandra Arrua Alvarenga

**Affiliations:** 1 Centro Multidisciplinario de Investigaciones Tecnológicas, Universidad Nacional de Asunción, Campus de la UNA, San Lorenzo 111421, Paraguay; 2 Facultad de Ciencias Agropecuarias, Unidad Pedagógica Hohenau, Universidad Católica Nuestra Señora de la Asunción, Hohenau 6290, Paraguay; 3 Facultad de Ciencias Agropecuarias y Forestales, Universidad Nacional de Itapúa, Encarnación 070102, Paraguay; 4 Facultad de Ciencias Exactas y Naturales, Universidad Nacional de Asunción, Campus de la UNA, San Lorenzo 111421, Paraguay; 5 Instituto de Investigaciones en Micología y Micotoxicología (IMICO), Consejo Nacional de Investigaciones Científicas y Técnicas-Universidad Nacional de Río Cuarto (CONICET-UNRC), Rio Cuarto 5800, Argentina; mramirez@exa.unrc.edu.ar (M.L.R.);

**Keywords:** plant pathogen, plant health surveillance, food safety

## Abstract

Maize (*Zea mays* L.) is a cornerstone of food security and livestock production in Paraguay. However, its productivity and grain safety are increasingly threatened by *Fusarium* species because of their pathogenic capacity and ability to produce mycotoxins. In this study, symptomatic maize leaves collected from commercial fields in Pirapó, Itapúa, during the 2022 growing season were processed to isolate and characterize fungal pathogens. Three isolates displaying typical *Fusarium* morphology were obtained and examined through macroscopic and microscopic traits. Molecular identification was conducted using translation elongation factor 1-α 1-α (*TEF*) sequences, followed by phylogenetic inference using maximum likelihood and Bayesian methods. The Paraguayan isolates (PYF-MZE22-01, -02, -03) clustered with the ex-type strain *Fusarium awaxy* CBS139380 in a strongly supported clade, confirming species identity. This finding constitutes the first record of *F. awaxy* associated with maize in Paraguay, thereby expanding its known geographical distribution. Considering that members of the *Fusarium fujikuroi* species complex are recognized producers of regulated mycotoxins, the detection of *F. awaxy* raises concerns regarding its pathogenic potential and possible implications for food safety. These results underscore the importance of integrating molecular diagnostics, toxigenic profiling, and surveillance programs to monitor emerging *Fusarium* taxa in South American agroecosystems.

## 1. Introduction

*Zea mays* L., commonly known as maize, plays a key role in global agriculture due to its adaptability, high yield, and economic importance [1]. In 2023, worldwide maize production reached approximately 1.24 billion tons from over 200 million hectares [2]. In Paraguay, maize covers more than 750,000 hectares and produces around 3.7 million tons annually, making it a strategic crop not only for human consumption but also as a primary resource for livestock production [3]. Within Paraguayan livestock systems, maize (*Zea mays* L.) serves as a strategic resource for livestock production, particularly during the dry season, when the growth and nutritional quality of natural pastures decline sharply. Beyond its role as grain for concentrating feed, maize is widely used as silage, and by incorporating leaves and stalks, it provides an essential source of energy and fiber for both beef and dairy cattle [4]. This relevance underscores the need to better understand the threats posed by fungal pathogens that compromise yield, grain quality, and food safety. Among these pathogens, species of the genus *Fusarium* stand out for their ubiquity, high diversity, and significant economic impact [5]. They can persist as both epiphytic and endophytic, invade the root system, and colonize xylem vessels, where the proliferation of hyphae causes vascular obstruction and interferes with the ascent of xylem sap to aerial tissues [5,6,7]. With over 400 genetically distinct species, many forming morphologically similar complexes, the genus *Fusarium* presents considerable identification challenges [8,9,10,11,12]. It belongs to the phylum Ascomycota (class *Sordariomycetes*, order *Hypocreales*, family *Nectriaceae*), is widely distributed worldwide, and is distinguished by its remarkable species diversity and economic relevance in agricultural systems [9,11,13,14]. *Fusarium* species exhibit high morphological variability, including distinct conidial forms, pigmentation types, septation characteristics, variations in reproductive structures, and substantial phylogenetic diversity, as revealed by multilocus sequence analyses [9,12,15]. The high variability within the genus has driven repeated taxonomic revisions and revealed previously overlooked hidden species [11,14]. Species complexes often share similar ecological traits and disease-causing abilities, affecting a broad range of hosts and environments, making it difficult to reliably distinguish species and to design effective, species-specific control approaches [9,14,16,17]. Mycotoxins are chemically diverse, low-molecular-weight secondary metabolites (generally 200–500 Da) synthesized by filamentous fungi that exert a wide range of toxicological effects on animals and humans [18,19,20,21]. Within the broad spectrum of *Fusarium* secondary metabolites, only three groups are consistently detected at levels relevant to food safety and animal health, which has led to the establishment of international regulatory limits for fumonisins (FUMs), trichothecenes (TRIs), and zearalenone (ZEA) [22]. Among them, the trichothecene analog deoxynivalenol (DON) and its derivatives are considered the most economically significant owing to their high prevalence and frequent association with yield and quality losses [23,24]. Several other metabolites, including beauvericin (BEA), enniatins (ENNs), fusaproliferin (FUP), fusaric acid (FA), fusarins (FUSs), and moniliformin (MON), have demonstrated toxicological effects under laboratory conditions, but lack confirmed links to natural outbreaks of mycotoxicoses [16,22,25]. Recent phylogenetic studies have revealed an increasing number of new and cryptic *Fusarium* species, many of which pose emerging threats to global agriculture [9,16]. The introduction of such species into new agroecological zones, facilitated by global trade, seed movement, and shifting climatic conditions, has the potential to modify local disease dynamics, expand the diversity of mycotoxins in staple grains, and undermine current management strategies [16,26,27]. The emergence of *F. awaxy* in maize is a novel and significant finding with both phytopathological and food safety implications. Unlike the more extensively studied members of the *F. fujikuroi* Species Complex, *F. awaxy* has only recently been reported in association with cereal crops, reflecting the expanding diversity of cryptic taxa within this group [28,29]. Its detection in Paraguayan maize fields is particularly relevant given the crop’s central role in national food security and agro-export markets. Considering that species within this complex are well-known producers of regulated mycotoxins and are adapted to diverse agroecological conditions [17,30], the occurrence of *F. awaxy* raises critical questions regarding its pathogenicity, toxigenic potential, and epidemiological dynamics in subtropical production systems. This highlights the urgent need for integrated surveillance and molecular characterization to predict its impact on maize health and food safety [31]. In Paraguay, research on *Fusarium* diversity in maize has not yet been conducted, and no studies have addressed the occurrence, taxonomy, or pathogenic potential of these species in local production systems. Therefore, the diversity of *Fusarium* species affecting maize in the country remains underexplored, and no records of *Fusarium awaxy* are available to date. Given the strategic importance of maize for both human consumption and livestock feeding systems in Paraguay, the emergence of new *Fusarium* species represents a significant concern because of their capability to produce toxic secondary metabolites. This study provides the first evidence of *F. awaxy* in maize from Paraguay based on phylogenetic analyses of EF-1α sequences, contributing to the understanding of local fungal diversity and highlighting the need for strengthened diagnostic, monitoring, and management strategies to safeguard agricultural productivity and food security.

## 2. Materials and Methods

### 2.1. Sample Collection

Between October and December 2022, during a field evaluation of fungicide performance, we opportunistically collected leaf samples from commercial maize (*Zea mays* L.) hybrids grown in production fields in Pirapó, Itapúa, Paraguay (−26.955742, −55.391232). Sampling targeted plants with necrotic foliar lesions. Across the surveyed plots, disease incidence was 100%, and lesion severity ranged from 30% to 60%, estimated using the 0–6 scale of Hernández-Ramos and Sandoval-Islas [32]. The etiology of the lesion was not determined in situ and may involve multiple agents. Five plants were sampled from each hybrid, and three symptomatic leaves per plant were collected. Samples were placed in sterile paper bags, transported on ice, and stored at −20 °C until processing immediately.

### 2.2. Surface Disinfection and Isolation

Five leaf fragments (~5 × 5 mm) were excised from the margins of necrotic lesions, surface-disinfected by immersion in 3% sodium hypochlorite (1 min), followed by 70% ethanol (1 min), and rinsed three times in sterile distilled water under agitation. Disinfected asymptomatic leaves from the same plants were processed under the same conditions as symptomatic samples, and plates containing only sterile PDA medium were used as environmental controls, which showed no fungal growth. These results indicate that cross-contamination did not occur during isolation, and contamination is considered unlikely since *F. awaxy* has never been handled in our laboratory and this study represents its first confirmed record in Paraguay. The fragments were blotted dry on sterile filter paper and plated on Potato Dextrose Agar. Plates were incubated at 25 ± 2 °C in the dark for 7 days, and fungal colonies emerging from the tissues were subcultured on fresh potato-dextrose-agar (PDA) medium to obtain pure cultures. Subsequently, monosporic cultures were established from the previously purified isolates [14].

### 2.3. Morphological Identification

Monosporic isolates were examined macroscopically for colony morphology (growth rate, pigmentation, and aerial mycelium) and microscopically for conidial characteristics. Observations of macroconidia, microconidia, and conidiogenous cells. Morphological identification at the genus level was conducted following the taxonomic keys of Leslie and Summerell [11] and Crous et al. [14]. For macromorphological characterization, colonies were grown on Potato Dextrose Agar (PDA) and incubated in darkness at 25 °C for 14 days. For micromorphological observations, cultures on Carnation Leaf Agar (CLA) were incubated under continuous near-UV light (24 h photoperiod) at 25 °C for 14 days [11,14].

Representative isolates were preserved on PDA slants at 4 °C for short-term storage and as glycerol stocks (20%) at −80 °C for long-term maintenance.

### 2.4. DNA Isolation, Amplification and Sequencing

Each strain was grown on PDA for one week at 22 ± 1 °C. The resulting mycelia were harvested by plate surface scraping, stored frozen at −20 °C until ground, and extracted using the CTAB method [11,33,34] to obtain fungal DNA. Its quality was analyzed by electrophoresis, quantified using a spectrophotometer (DS-11-DeNovix, Wilmington, DE, USA) [35], and stored at −25 °C in a freezer. Amplification of the translation elongation factor 1-α (*TEF1*) gene was carried out with PCR primers EF1 and EF2 using the amplification conditions described by O’Donnell et al. (1998) [36]. PCR reactions were carried out on a thermal cycler MJ Research PTC-200 (MJ Research Inc., Watertown, MA, USA) and the reaction conditions were: denaturation at 94 °C for 1 min; 34 cycles of the denaturation at 94 °C for 30 s, annealing at 56 °C for 45 s, extension at 72 °C for 1 min; final extension at 72 °C for 5 min, followed by cooling at 4 °C to develop the next step. Agarose gels of 0.8% concentration were prepared in 0.5× TBE (Tris borate EDTA) buffer and run at 90 V and 100 mA for 60 min. Amplification products were compared to a 100 bp molecular weight marker, EZ Load™ 100 bp Molecular Ruler (#170- 8352), BioRad, Hercules, CA, USA [37]. For gel staining, 1× Diamond red intercalator Diamond™ Nucleic Acid Dye (Promega), Madison (WI), USA was used [38]. The gels were visualized using a Gel Documentation System with UV light in Gel Doc EZ–BioRad, BioRad, Hercules, CA, USA [39]. PCR products were purified and sequenced by Macrogen, Inc. (Seoul, South Korea) using the same primers used for PCR amplification. Sequences were edited with BioEdit Sequence Alignment Editor 7.1.3.0 (North Carolina State University, Raleigh, (NC), USA) [40] and compared with FUSARIUM-ID [41] and GenBank databases for the identification of the isolates.

### 2.5. Phylogenetic Analyses

According to BLAST v2.16.0., Bethesda, Maryland (MD), USA. searches, the three analyzed sequences were aligned with *F. awaxy* reference sequences downloaded from the National Center for Biotechnology Information (NCBI). The three strains and the other 26 species within the American clade of the *Fusarium fujikuroi* species complex (FFSC) were used in the phylogenetic analysis, with *F. oxysporum* NRRL 22902 (GenBank *TEF1* accession number: AF160312) as the outgroup (Table 1). The nucleotide sequences of the *TEF1* amplicons were aligned using MAFFT online version 7 [42]. Aligned sequences were subjected to Bayesian phylogenetic inference (BI) using MrBayes 3.2.6 [43] and Maximum likelihood (ML) analysis using PhyML 3.1 [44]. For both BI and ML analyses, the best substitution model was determined using jModelTest [45] and scored following the Akaike information criterion (AIC). The General Time-Reversible (GTR) substitution model + gamma-distributed rate variation across sites (G) was used. Two runs with four chains each were run for ten million generations, with a sampling frequency of every 100 generations. Trees after the initial 25% trees of each run were discarded as burn-in. Tree topologies were adjusted using FigTree v1.4.3. The DNA sequences generated in this study were deposited in GenBank under accession numbers (Table 1).

## 3. Results

### 3.1. Culture Characteristics

**Figure 1:**
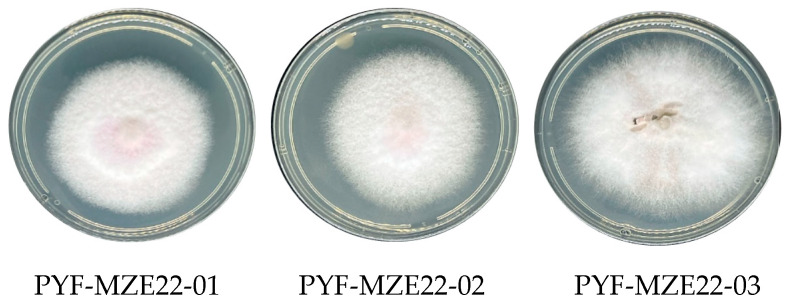
Colonies of *Fusarium awaxy* grown on Potato Dextrose Agar (PDA) and incubated in the dark for 15 days at 24 ± 2 °C.

Three isolates that exhibited morphological characteristics consistent with those of *Fusarium* were obtained from the processed samples. Pure cultures were evaluated based on macroscopic traits, including growth rate, pigmentation, and aerial mycelium development, as well as microscopic features, focusing on conidial morphology [14]. Culture characteristics: Colonies on PDA incubated in the dark displayed an average radial growth rate of 5.6 mm/day at 24 ± 2 °C, reaching 75–85 mm in diameter within 15 days (Figure 1). Colonies produced abundant aerial mycelium and were initially white, pale pink, and pale violet in older cultures [46]. On CLA microconidia forming in false heads in aerial mycelium, arising in monophialides and polyphialides, macroconidias 3-septate, 29.8–56.6 μm large ($\bar{x}$ = 38.2 μm) and 4.0–6.6 μm wide ($\bar{x}$ = 4.1 μm wide). Microconidias 10.3–19.7 μm large ($\bar{x}$ = 13.8 μm) and 3.0–5.7 μm wide ($\bar{x}$ = 3.9 μm wide). *Chlamydospores* absent. No odor was detected.

### 3.2. Phylogenetic Analysis

**Figure 2:**
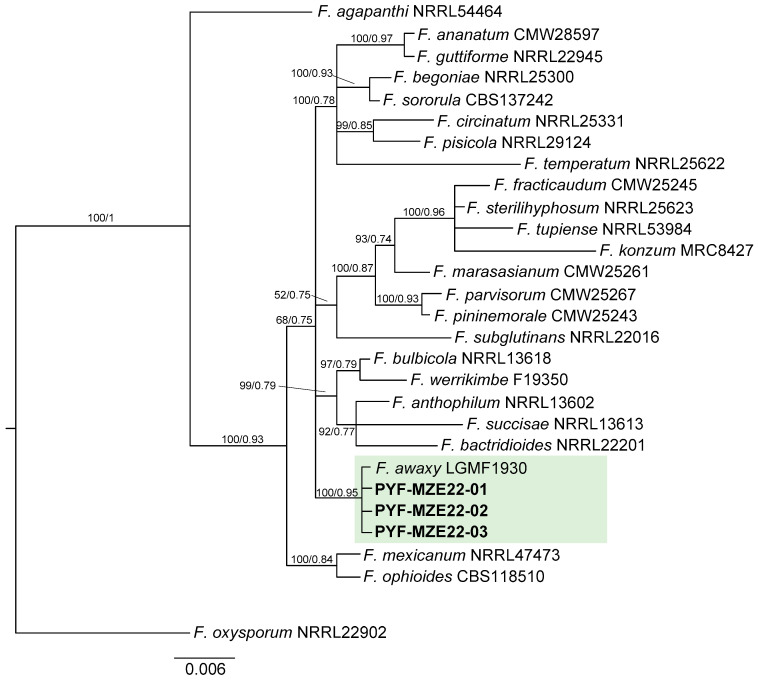
Bayesian inferred tree based on partial sequences of the *TEF1* gene. Values at branch nodes indicate branch support with posterior probabilities (PP × 100); values ≥ 50 are shown. Both Bayesian Inference (BI) and Maximum likelihood (ML) analysis yielded a tree with the same topology. The tree was rooted with a sequence from *F. oxysporum* NRRL 22902.

The *TEF1* sequence data set consisted of 30 sequences of a 600-base alignment. In the BI and ML analysis of *TEF1* sequences, the three Paraguayan isolates from this study formed a well-supported monophyletic clade that included *F. awaxy* LGMF 1930 reference strain (Figure 2). Both BI as well as ML trees exhibited the same topology. The robustness of ML and BI analyses allowed the identification of the strains isolated from the present study as *F. awaxy* (Figure 2).

## 4. Discussion

This study is the first to report the presence of *F. awaxy* isolated from maize in Paraguay, thus expanding the known geographical distribution of this recently described species. However, recent studies have shown that cryptic species and overlapping morphological traits often complicate field-level identification, emphasizing the importance of multilocus phylogenetics and genome-scale approaches for accurate species delimitation [9]. The detection of *F. awaxy* aligns with recent findings in Brazil from rotten stalks [46], South Africa from white and yellow maize kernels collected post-harvest but pre-storage [28], and the United States from ears, stalks, and roots [31]. In China, *F. awaxy* was detected as a pathogen causing maize stalk rot [17,29], where the species was also reported for the first time in maize, revealing highly diverse *Fusarium* communities associated with cereals, and where the species has been detected in maize, highlighting its emerging role in diverse agroecosystems.

The emergence of *F. awaxy* in South America has phytopathological and food safety implications because maize is highly susceptible to *Fusarium* infections and the associated risks [5]. Although the toxigenic profile of *F. awaxy* remains poorly characterized [17,28,29,30,46], fumonisin production in grains and plant tissues has been documented [28,30]. These observations warrant further targeted fumonisin production characterization of the species. The occurrence of fumonisins in agricultural products depends on environmental and post-harvest factors, including region, season, and storage conditions [19].

Fumonisin exposure is recognized as a major health concern for humans and animals because these mycotoxins interfere with key cellular mechanisms and contribute to diverse toxic and pathological outcomes [47]. Human exposure to fumonisins has been associated with an elevated risk of esophageal and hepatic cancers, as well as developmental abnormalities such as neural tube defects, highlighting their relevance as public health hazards [48,49]. In animals, fumonisin exposure induces a variety of species-specific toxicoses. In equids, it causes leukoencephalomalacia [50] due to disruption of sphingolipid metabolism, while in swine, it leads to pulmonary edema and cardiopulmonary failure [19,51]. In cattle and other ruminants, the ingestion of contaminated feed has been associated with feed refusal, hepatocellular degeneration, and severe renal tubular necrosis [19,52]. In poultry, fumonisins have been implicated in acute broiler mortality syndrome, which is characterized by increased embryonic mortality and high death rates in young broilers [19,53,54]. Members of the *F. fujikuroi* and *F. sambucinum* complexes are well-known producers of fumonisins, trichothecenes, and zearalenone [22]. The emergence of *F. awaxy* in Paraguay parallels the trends observed in Africa and Asia, suggesting that environmental changes may facilitate the spread of cryptic or newly described taxa across continents [17,55,56]. However, mycotoxin occurrence does not always correlate with the presence of specific *Fusarium* species [10,28], reflecting the need for further chemical and genomic characterization of Paraguayan isolates. The detection of multiple strains in a limited area suggests that *F. awaxy* may be more widespread than previously recognized [9,28].

Our findings highlight the emergence of novel *Fusarium* taxa in South American agroecosystems. The detection of *F. awaxy* in Paraguayan maize raises concerns regarding plant health, food safety, and trade. This study was based on a limited number of samples, from which only three *F. awaxy* isolates were obtained. Consequently, statistical analyses, such as replication-based inference or estimation of variability, were not applicable. Therefore, the findings should be interpreted within the scope of an initial exploratory assessment that confirms the occurrence of *F. awaxy* in maize in Paraguay. Future studies should include broader sampling and quantitative analyses to deepen our understanding of the species’ biology, distribution, and toxigenic potential.

## 5. Conclusions

This is the first study to report the presence of *F. awaxy* associated with maize in Paraguay. Phylogenetic analyses based on *TEF1* sequences confirmed the identity of three isolates obtained from symptomatic plants. The detection of this species expands its known geographical distribution and highlights the need to strengthen monitoring and diagnostic efforts to anticipate its potential impact on maize production and food safety in Paraguay.

## Figures and Tables

**Table 1:** *Fusarium* species included in this study.

*Fusarium* Species	Strain Number	Host	Origin	*TEF1* GenBank Accession Number
*F. agapanthi*	NRRL 54463	*Agapanthus praecox*	Australia	KU900630
*F. ananatum*	CMW 28597	*Ananas comosus*	South Africa	EU668312
*F. anthophilum*	NRRL13602	*Hippeastrum* sp.	Germany	AF160292
*F. awaxy*	LGMF 1930	Rotten stalk of *Zea mays*	Brazil	MG839004
*F. awaxy*	PYF-MZE22-01	*Zea mays*	Paraguay	PX214124
*F. awaxy*	PYF-MZE22-02	*Zea mays*	Paraguay	PX214125
*F. awaxy*	PYF-MZE22-03	*Zea mays*	Paraguay	PX214126
*F. bactridioides*	NRRL 22201	*Cronartium conigenum*	USA	KC514053
*F. begoniae*	NRRL 25300	*Begonia elatior*	Germany	AF160293
*F. bulbicola*	NRRL 13618	*Nerine bowdenii*	Germany	AF160294
*F. circinatum*	NRRL 25331	*Pinus radiata*	USA	AF160295
*F. fracticaudum*	CMW 25245	*Pinus maximinoii*	Colombia	KJ541059
*F. guttiforme*	NRRL 22945	*Ananas comosus*	England	AF160297
*F. konzum*	MRC 8427	*Sorghastrum nuttans*	USA	LT996098
*F. marasasianum*	CMW 25261	*Pinus patula*	Colombia	KJ541063
*F. mexicanum*	NRRL 47473	*Mangiferia indica*	Mexico	GU737416
*F. ophioides*	CBS 118510	*Panicum maximum*	South Africa	MN534020
*F. oxysporum*	NRRL22902	*Pseudotsuga menziesii*	USA	AF160312
*F. parvisorum*	CMW 25267	*Pinus patula*	Colombia	KJ541060
*F. pininemorale*	CMW 25243	*Pinus tecunumanii*	Colombia	KJ541064
*F. pilosicola*	NRRL 29124	*Bidens pilosa*	USA	MN534055
*F. sororula*	CBS 137242	*Pinus patula*	Colombia	KJ541067
*F. sterilihyphosum*	NRRL 25623	*Mango*	South Africa	AF160300
*F. subglutinans*	NRRL 22016	*Zea mays*	USA	AF160289
*F. succisae*	NRRL 13613	*Succisa pratensis*	Germany	AF160291
*F. temperatum*	NRRL25622	Zea mays	South Africa	AF160301.1
*F. tupiense*	NRRL 53984	*Magnifera indica*	Brazil	GU737404
*F. werrikimbe*	F19350	*Sorghum leiocladum*	Australia	EF107131

## Data Availability

The original data presented in the study are openly available in NCBI; accession numbers are PX214124, PX214125, and PX214126.

## Abbreviations

The following abbreviations are used in this manuscript:

PDA	Potato Dextrose Agar
CLA	Carnation Leaf Agar
